# TGF-β Negatively Regulates CXCL1 Chemokine Expression in Mammary Fibroblasts through Enhancement of Smad2/3 and Suppression of HGF/c-Met Signaling Mechanisms

**DOI:** 10.1371/journal.pone.0135063

**Published:** 2015-08-07

**Authors:** Wei Bin Fang, Benford Mafuvadze, Min Yao, An Zou, Mike Portsche, Nikki Cheng

**Affiliations:** Department of Pathology and Laboratory Medicine, University of Kansas Medical Center, Kansas City, KS, United States of America; Baylor College of Medicine, UNITED STATES

## Abstract

Fibroblasts are major cellular components of the breast cancer stroma, and influence the growth, survival and invasion of epithelial cells. Compared to normal tissue fibroblasts, carcinoma associated fibroblasts (CAFs) show increased expression of numerous soluble factors including growth factors and cytokines. However, the mechanisms regulating expression of these factors remain poorly understood. Recent studies have shown that breast CAFs overexpress the chemokine CXCL1, a key regulator of tumor invasion and chemo-resistance. Increased expression of CXCL1 in CAFs correlated with poor patient prognosis, and was associated with decreased expression of TGF-β signaling components. The goal of these studies was to understand the role of TGF-β in regulating CXCL1 expression in CAFs, using cell culture and biochemical approaches. We found that TGF-β treatment decreased CXCL1 expression in CAFs, through Smad2/3 dependent mechanisms. Chromatin immunoprecipitation and site-directed mutagenesis assays revealed two new binding sites in the CXCL1 promoter important for Smad2/3 modulation of CXCL1 expression. Smad2/3 proteins also negatively regulated expression of Hepatocyte Growth Factor (HGF), which was found to positively regulate CXCL1 expression in CAFs through c-Met receptor dependent mechanisms. HGF/c-Met signaling in CAFs was required for activity of NF-κB, a transcriptional activator of CXCL1 expression. These studies indicate that TGF-β negatively regulates CXCL1 expression in CAFs through Smad2/3 binding to the promoter, and through suppression of HGF/c-Met autocrine signaling. These studies reveal novel insight into how TGF-β and HGF, key tumor promoting factors modulate CXCL1 chemokine expression in CAFs.

## Introduction

Fibroblasts are a key cell type found in connective tissues throughout the body, and regulate multiple biological processes including inflammation, wound healing and tumor progression [1–3]. Distinguished by their spindle cell morphology, fibroblasts are identified by expression of mesenchymal markers such as vimentin, fibroblast specific protein and desmin [3]. In breast cancer, the accumulation of fibroblasts correlates with invasive cancer progression and poor patient prognosis [3, 4]. Co-transplantation of carcinoma associated fibroblasts (CAFs) with breast carcinoma cells in rodents results in increased tumor growth, survival and metastasis [5–7]. Breast tumor outgrowth is inhibited by co-transplantation of normal fibroblasts (NAFs) [8, 9]. NAFs and CAFs appear similar in cell morphology; however, gene profiling studies suggest that CAFs show increased expression of tumor promoting factors, such as growth factors and cytokines [10–12]. CXCL1 is one such factor.

CXCL1 is a small soluble molecule (8kda) belonging to the family of molecules known as chemokines. CXCL1 normally regulates recruitment of bone marrow derived cells during wound healing and infection [13–15]. While CXCL1 expression is de-regulated in a number of solid tumors, including: melanoma, prostate cancer, bladder cancer, CXCL1 has been shown to play important functional roles in breast tumors [15, 16–18]. CXCL1 promotes breast tumor growth, metastasis and chemo-resistance through recruitment of Gr1+ myeloid cells, and by directly signaling to cancer cells [19]. In recent studies, we demonstrated that CXCL1 was overexpressed in breast cancer stroma, and correlated with increased disease recurrence, and decreased overall survival [20]. Increased expression of CXCL1 in breast cancer stroma inversely correlated with expression of Transforming Growth Factor-beta (TGF-β) signaling proteins. CXCL1 expression was increased in cultured fibroblasts that expressed low levels of TGF-β [20]. These studies suggested an inverse relationship between CXCL1 expression and the TGF-β signaling pathway.

TGF-β is a cytokine that plays important roles in in the regulating the growth and activity of fibroblasts. TGF-β functions by signaling to cell surface type II receptors, which recruit type I receptors, resulting in the activation of downstream signaling cascades including canonical Smad pathways, to modulate gene transcription [21, 22]. TGF-β signaling in fibroblasts functions to modulate expression of tissue remodeling factors, including extracellular matrix proteins, proteases and angiogenic factors [23, 24]. Interestingly, co-transplantation of TGF-β signaling deficient fibroblasts with mammary carcinoma cells in nude mice enhanced tumor growth and invasion, and increased growth factor receptor tyrosine kinase signaling in cancer cells [25, 26]. These studies indicate that TGF-β signaling in mammary fibroblasts functions to suppress tumor progression by negatively regulating expression of oncogenic signaling factors.

Given the inverse relationship between TGF-β signaling and CXCL1 expression in CAFs, we hypothesized that TGF-β negatively regulates CXCL1 expression in CAFs. Using siRNA and pharmacologic approaches on cultured cells, we demonstrate that TGF-β inhibits CXCL1 expression in CAFs through Smad2/3 binding to the promoter. Furthermore, TGF-β inhibits CXCL1 expression through a secondary mechanism that involves the suppression of HGF/c-Met autocrine signaling. These studies reveal novel insight into how TGF-β and HGF, key factors that are expressed in breast tumors, coordinate CXCL1 chemokine expression in CAFs.

## Materials and Methods

### Cell Culture

Murine fibroblast lines (41CAF, 83CAF, 311NAF) were isolated and characterized in previous studies [20, 27, 28]. Briefly, 41CAFs and 83CAFs were isolated from transgenic mice (FVB) expressing the PyVmT oncogene under the control of the Mouse Mammary Tumor Virus Promoter (MMTV), at 12–16 weeks of age. Normal mammary fibroblasts (311NAF) were isolated from the mammary glands of wild-type C57BL/6 mice at 12–16 weeks of age. Human cancer associated fibroblasts were isolated from patient specimens of invasive breast ductal carcinoma, using methods previously described [27]. 4T1 mammary carcinoma cells were generously provided by Fred Miller (University of Michigan, Ann Arbor, MI). All cell lines were cultured on plastic in DMEM media containing 10% FBS with 0.1% amphotericin, 1% penicillin-streptomycin (Cellgro).

### siRNA Transfection

Cells were seeded in 24-well plates at a density of 20,000 cells/well. For each well, 24 pmol control siRNAs or siRNAs targeting Smad2, Smad3 and HGF (Santa Cruz Biotechnology were complexed to 2.4 μl lipofectamine 2000 (Invitrogen) in 100 μl Opti-MEM medium for 20 minutes at room temperature. Cells were incubated in 400 μl Opti-MEM with the siRNA/lipofectamine complexes for 24 hours, and recovered in Opti-MEM/10% medium for an additional 24 hours. Cells were then incubated for 24 hours in 500 μl Opti-MEM for HGF ELISA or in Opti-MEM/10% FBS in the presence or absence of TGF-β for ELISA, luciferase or immunoblot analysis.

### ELISA

Cells were seeded in 24-well plates at a density of 20,000 cells/well. To generate conditioned medium, cells were incubated in 500 μl Opti-MEM (Gibco) media for 24 or 48 hours in the presence of absence of HGF (Peprotech), c-Met kinase inhibitor II (Calbiochem), SN50 (Calbiochem) or Bay 11–7085 (Enzo Life Sciences cat. no. BML-EI279-010). Cells were treated with TGF-β for 24 or 48 hours in Opti-MEM containing 10% FBS. Murine HGF expression was analyzed in 100 μl of conditioned medium according to manufacturer protocol (R&D Systems). Murine or human CXCL1 expression was analyzed in 20 μl conditioned medium diluted in 80 μl Opti-MEM media, according manufacturer’s instructions (Peprotech). Reactions were catalyzed using tetramethylbenzidine substrate (Thermo Scientific). Reaction was stopped using 50 μl/well of 2N HCl, and read at *A*450nm using a Biotek plate reader.

### Luciferase Assay

The firefly luciferase reporter containing the CXCL1 promoter region -701 to +30 (PGL3.luc.CXCL1) was kindly provided by Katherine Roby, Ph.D (University of Kansas Medical Center, Kansas City, KS). The firefly luciferase reporter driven by NF-κB (pNFκB-luc) five copies of an enhancer element for NF-κB as described [29], and was obtained from Agilent Technologies (cat. no. 219078). The firefly luciferase reporter driven by pC/EBP-β (pC/EBP-β.luc,) contains the enhancer element CCAAT in triplicate, and was obtained from Agilent Technologies (cat. no. 240112). The 3TP-lux firefly luciferase reporter (Addgene) was generated as described [30]. The Renilla luciferase reporter plS2 (Addgene) was generated as described [31]. Cells were seeded in 6 cm cell culture dishes (Corning, Product #430166) at a density of 150,000 cells. For each well 8 μg firefly luciferase plasmids and 400 ng Renilla luciferase plasmids were complexed to 15 μl Lipofectamine LTX containing 8.4 μl Plus reagent (Invitrogen) for 20 minutes at room temperature in 500 μl Opti-MEM. Cells were incubated with plasmid/lipofectamine complexes for 24 hours, recovered in Opti-MEM/10% FBS for an additional 24 hours, and then re-seeded into 24-well plates (20,000 cells/well), for siRNA transfection. Samples were harvested and analyzed using the Dual-Luciferase Reporter Assay system (Promega), according to manufacturer’s instructions. 20 μl lysates were assayed in triplicate using a Veritas Microplate Luminometer (Turner BioSystems).

### Immunoblot Analysis

Cells were rinsed with PBS twice, lysed in RIPA buffer containing 10 mM Tris-HCl, pH 8.0, 0.1 mM EDTA, 0.1% sodium deoxycholate, 0.1% SDS, and 140 mM NaCl, supplemented with a cocktail of protease and phosphatase inhibitors (Sigma) and 10 mM of sodium orthovanadate (Sigma). 80 μg of protein were resolved by 10% SDS-PAGE. The proteins were transferred to nitrocellulose membranes and then probed with antibodies (1:1000) to: Smad2/3 (BD Biosciences, mouse monoclonal, cat. no.610843), Smad3 (Cell Signaling Technology, rabbit monoclonal, cat. no. 9523), phospho-Smad2 (Ser-465/467, Cell Signaling Technology, rabbit polyclonal, cat. no.3101), phospho-Smad3 (Ser-423/425, Cell Signaling Technology, rabbit monoclonal, cat. no.9520), c-Met (Cell Signaling Technology, rabbit polyclonal, cat. no.4560), phospho-c-Met (Tyr-1234/1235, Cell Signaling Technology, rabbit monoclonal, cat. no.3077), or pan-actin (Cell Signaling Technology, rabbit monoclonal, cat. no.8456). Specific immunoreaction was detected with secondary goat anti-rabbit conjugated to horse radish peroxidase (hrp) (Rockland, cat. no. 611–1302) or goat anti-mouse-hrp (Biorad, cat. no. 170–5047) at a 1:1000 dilution, and incubation with West Pico ECL Western blotting substrate (ThermoScientific).

### Site-Directed Mutagenesis

Point mutations were introduced into the SBEs within the PGL3.luc.CXCL1 reporter by site-directed mutagenesis, using QuickChange Lightning Site-Directed Mutagenesis Kit (Agilent). The design of the Smad mutations was based on previous studies, which validated the function and specificity of the mutant SBEs towards Smad protein binding [32]. SBE1 (from -249 bp relative to TSS) was mutated from 5’-GTCTC-3’ to 5’-TGAGA-3’, using the following primers (5’ to 3’): SBEm1 sense: CCTGAGCACTGGAGACTCTGAATGAGAACTACTCCTCCCCCCCCCA, SBEm1 anti-sense: TGGGGGGGGGAGGAGTAGTTCTCATTCAGAGTCTCCAGTGCTCAGG; SBE2 (from -144bp relative to TSS) was mutated from 5’-GTCTA-3’ to 5’-TGAGC-3’ using the following primers: SBE2m1 sense: CCCCCTTGCTCCACTCCCAAGGATGCTCATCTGGGATTTTTGCTTTTTGCCCC, SBEm2 anti-sense: GGGGCAAAAAGCAAAAATCCCAGATGAGCATCCTTGGGAGTGGAGCAAGGGGG. Mutations into both SBEs (SBEm1/2) were introduced into a single construct by mutating SBE2 in the construct containing SBEm1. Mutations were validated by sequencing.

### Chromatin Immunoprecipitation Assay

The assay was adapted from previous studies [33]. Cells were cultured in 15 cm tissue culture dishes to 90% density (approximately 2 million cells/plate), and then synchronized in Opti-MEM overnight. Cells were treated with 5 ng/ml TGF-β for 6 hrs in Opti-MEM/10% FBS, fixed in 1% formaldehyde for 10 minutes. Samples were scraped into ice cold PBS containing protease inhibitors, centrifuged and re-suspended in 1.5 ml lysis buffer containing 50 mM Tris, pH 8.0, 1% SDS, 1% Triton X-100 and 10mM EDTA. DNA was fragmented to 200–1000 bp by sonicating 24 times for 10 sec each using a Vibra Cell Sonicator set at 42% output. Sonicated samples were incubated overnight at 4°C with 1.2 μg of antibody to Smad2 (Cell Signaling Technology, rabbit monoclonal, cat. no. 5339) or 0.96 μg Smad3 (Cell Signaling, cat. no. 9523) or rabbit IgG control (Cell Signaling Technology, rabbit monoclonal, cat. no. 2729). Samples were incubated with 30 μl of ChIP grade Protein G magnetic beads (Cell Signaling) at 4°C for 2 hrs. Beads were captured using a DynaMag-2 magnetic rack (Life Technologies) and washed in buffer containing 25 mM Tris, pH 8.0, 150 mM NaCl, 1% Triton X-100, 3 mM EDTA, 0.05% SDS. Samples were eluted with 400 μl of buffer containing 100 mM NaHCO_3_ and 1% SDS at 65°C for 15 minutes. Input controls were prepared by adding 350 μl of elution buffer and 16 μl of 5 M NaCl to 50 μl of sonicated lysates. Samples were de-crosslinked at 95°C for 30 min, and treated with 40 μg of RNAase (Thermo Scientific) at 37°C for 20 min. DNA was purified using QIAquick PCR purification kit (QIAGEN Science) according to manufacturer’s instructions.

The following primers (IDT Technologies) were synthesized for real time PCR: SBE1+SBE2 sense (5’ to 3’): CCTGAGCACTGGAGACTCTG, SBE1+SBE2 anti-sense (5’ to 3’): TGCTCCACTCCCAAGGATTA, SBE2 sense (5’ to 3’): CACTTGTCCAGCGAAGCAC, SBE2 anti-sense (5’ to 3’): GGAAATTCCCGGAGTACAGG, PAI sense (5’ to 3’): CAGTCATCTCAGGCTGCTGT, PAI anti-sense (5’ to 3’): GGCTCGCTCTTTGTGTCAAT. SBE1+SBE2 was designed to yield a 148 bp fragment containing both SBEs at -249 to -246 (SBE1) and -144 to -141 (SBE2), by amplifying the promoter region from -271 to -123 upstream of the TSS. SBE2 was designed to yield a 150 bp fragment by amplifying the region -212 to -62, containing SBE2 only. PAI primers were designed to yield a 163 bp fragment by amplifying the PAI1 promoter region at position -800 to -637.

PCR reactions were performed with 4 μl of sample at 95°C for 15 seconds, 60°C for 1 minute for 40 cycles using the StepOne System (Applied Biosystems). Samples were run in triplicate, and the signals were normalized to signals obtained from input control samples. The signals were normalized to signals obtained from input control samples.

### Immunohistochemistry/Immunofluorescence

Five micron sections were prepared from normal human breast or breast ductal carcinoma tissues or PyVmT mammary carcinoma tissues that were generated from previous studies [28]. Sections were de-waxed and rehydrated in PBS. Sections were subjected to antigen retrieval in 2 M Urea for 2 minutes at 100°C in a pressure cooker, and washed in PBS. Endogenous peroxidases were quenched in PBS containing 3% H_2_0_2_ and 10% methanol for 30 minutes. After rinsing in PBS, samples were blocked in PBS containing 5% fetal bovine serum and incubated with c-Met antibodies (Cell Signaling Technologies, cat. no.4560) or α-smooth muscle actin antibodies (Abcam, cat no. 7817) at a 1:100 dilution overnight at 4°C. For immunohistochemistry staining, samples were washed in PBS, incubated with anti-rabbit biotinylated antibodies (Vector Labs, cat. no. BA 1000) at a 1:500 dilution, conjugated with streptavidin peroxidase (Vector Labs), and incubated with 3,3'-Diaminobenzidine (DAB) substrate (Vector Labs). Sections were counterstained with Harris’s hematoxylin for 5 minutes, dehydrated and mounted with Cytoseal. To detect c-Met expression by immunofluorescence, sections were incubated with secondary anti-rabbit antibodies conjugated to Alexa-568 (Invitrogen, cat. no. A10042) at a 1:500 dilution. To detect α-sma expression by immunofluorescence, sections were incubated with secondary anti-mouse-biotinylated antibodies (Vector Labs, cat. no BA9200) at a 1:500 dilution, and streptavidin conjugated to Alexa-488 (Invitrogen cat. no.S32354). Sections were counterstained with DAPI and mounted with ProLong anti-Fade (Invitrogen, cat. no. P3694).

### Ethics Statement

The breast tissues were obtained through a human subjects exemption, which was assigned by the Human Research Protection Program (ethics committee) at the University of Kansas Medical Center (#080193). The tissues were collected by the Biospecimen Respository Core Facility (BRCF), an IRB approved core facility, which had obtained written informed consent from patients. Tissue samples were de-identified by the BRCF prior to distribution. Existing medical records were used in compliance with institutional regulations. These regulations are aligned with the World Medical Association Declaration of Helsinki.

### Statistical Analysis

Experiments were performed in a minimum of triplicate. Values are expressed as Mean ± Standard Error of the Mean (SEM). Statistical analysis was performed using Two-tailed student T-Test or One-Way ANOVA with Bonferroni post-hoc analysis. Statistical significance was determined p-value less than 0.05; *p<0.05, **p<0.01, ***p<0.001, n.s; not significant or p≥0.05.

## Results

### Smad2 and Smad3 Are Required for TGF-β Mediated Suppression of CXCL1 Expression

**Fig 1:**
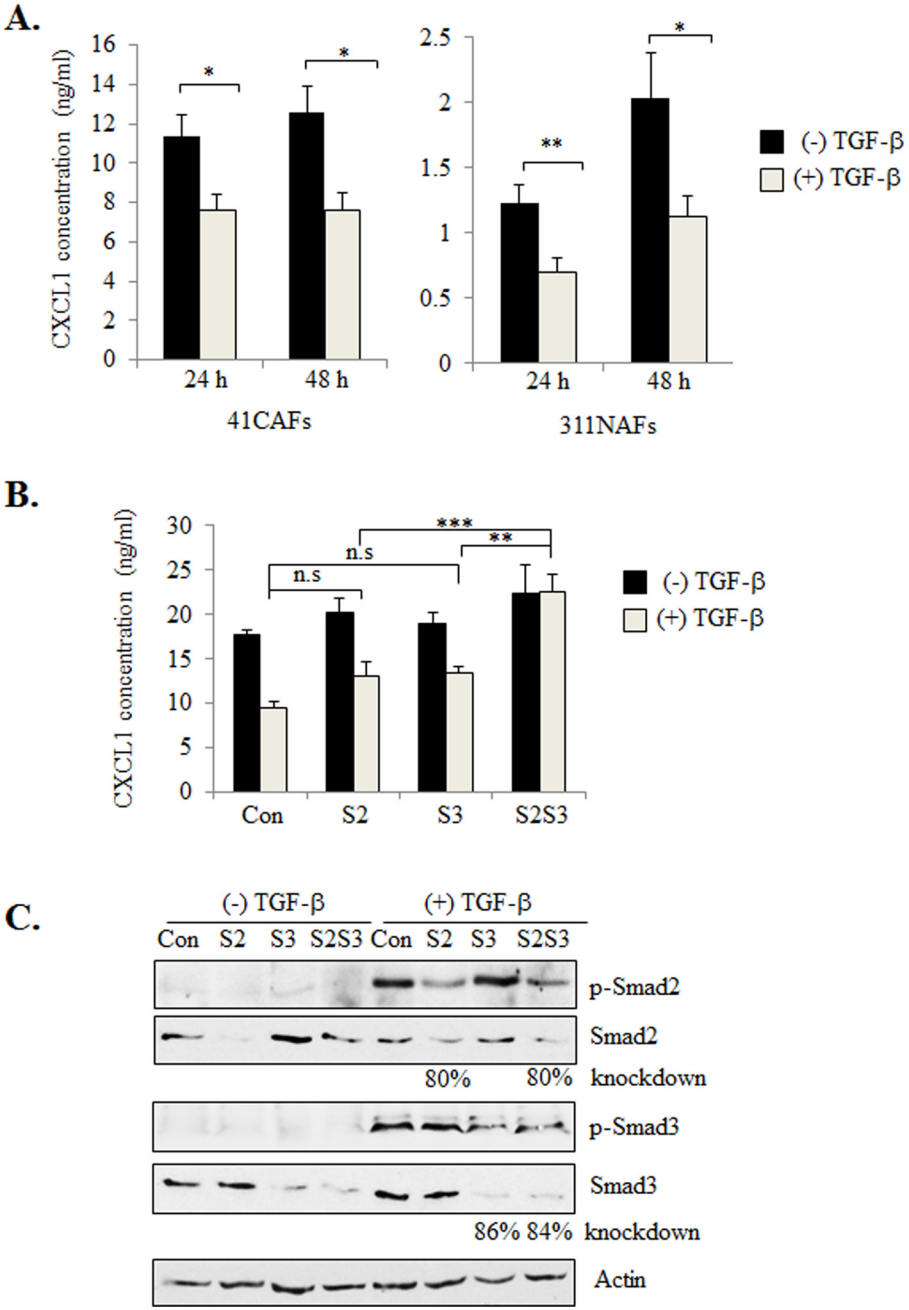
Knockdown of Smad2 and Smad3 counteracts TGF-β mediated suppression of CXCL1 expression. (A) 41CAFs and 311NAFs were treated with 5 ng/ml of TGF-β for 24 hours and 48 hours and analyzed for CXCL1 expression by ELISA. (B) 41CAFs were transfected with control siRNA (Con), siRNAs to Smad2 (S2), Smad3 (S3) or both (S2S3), treated with TGF-β for 24 hours, and analyzed for CXCL1 expression by ELISA. (C) 41CAFs transfected with siRNA were treated with TGF-β for 1 hour, and analyzed by western blot for expression of the indicated proteins. Expression levels of Smad2 and Smad3 were normalized to actin through densitometry analysis. Statistical analysis was performed using Two Tailed T-test (A), or One Way ANOVA followed by Bonferonni post-hoc comparisons (B). Statistical significance was determined by p<0.05; *p<0.05, **p<0.01, ***p<0.001, n.s; not significant. Values are expressed as Mean ± SEM.

We had previously demonstrated that the murine CAF lines 41CAFs and 83CAFs, expressed higher levels of CXCL1 expression and lower levels of TGF-β compared to normal mammary fibroblasts (311NAF), similar to expression patterns of human breast cancer stroma [28]. These studies demonstrated that the mouse fibroblast lines were reliable models to examine the molecular mechanisms regulating CXCL1 expression. To determine the importance of TGF-β in regulating CXCL1 expression in stromal cells, 41CAFs and 311NAFs were treated with TGF-β and examined for CXCL1 expression over time. By ELISA, TGF-β significantly decreased CXCL1 expression in 41CAFs and 311NAFs after 24 and 48 hours (Fig 1A).

To determine the downstream effectors that were involved in TGF-β mediated suppression of CXCL1, we screened for candidate pathways regulated by TGF-β. Activation of the Smad pathway is a key mechanism through which TGF-β signaling regulates cell proliferation, differentiation and EMT. TGF-β receptor activation leads to the recruitment and phosphorylation of Smad2 and Smad3 proteins, which complex with Smad4 proteins. These complexes translocate to the nucleus to regulate transcription of genes necessary for fibroblast growth and promotion of cancer cell invasion including: extracellular matrix proteins, proteases and cell cycle proteins [34]. Given the importance of Smad2 and Smad3 in modulating TGF-β responsiveness, 41CAFs were transfected with control siRNAs or siRNAs to Smad2 or Smad3, or both, and analyzed for CXCL1 expression by ELISA. There was no significant difference in CXCL1 expression with either Smad2 or Smad3 knockdown, regardless of TGF-β treatment. Knockdown of both Smad2 and Smad3 significantly increased CXCL1 expression in TGF-β treated cells (Fig 1B). By western blot analysis, transfection of siRNAs to Smad2 or Smad3 in 41CAFs decreased expression of Smad2 by 80% or Smad3 by 86%, and reduced expression of phosphorylated proteins (Fig 1C), demonstrating efficiency of siRNA knockdown. siRNA knockdown of both Smad2 and Smad3 in another murine CAF cell line, 83CAF, also inhibited TGF-β responsiveness and increased CXCL1 expression (S1 Fig). In summary, these studies indicate that both Smad2 and Smad3 modulate TGF-β suppression of CXCL1 in mammary CAFs.

### TGF-β Enhances Smad2 and Smad3 Binding to CXCL1 Promoter Elements

While Smad2 and 3 proteins function as transcriptional regulators with DNA binding capacity, Smad2/3 binding to the CXCL1 promoter had not been previously characterized. We searched the CXCL1 promoter upstream of the transcriptional start site (TSS) for known TGF-β inhibitory elements (TIE; 5’-GGCTT-3’) [35], or canonical Smad binding elements (SBEs), which would enable Smad2/3 proteins to interact with and suppress nearby co-factors [36, 37]. While known TIEs were not detected, two SBEs with the sequence: 5’-GTCT-3’[38, 39] were identified at -249 to -246 (SBE1) and -144 to -141 (SBE2), relative to the TSS.

**Fig 2:**
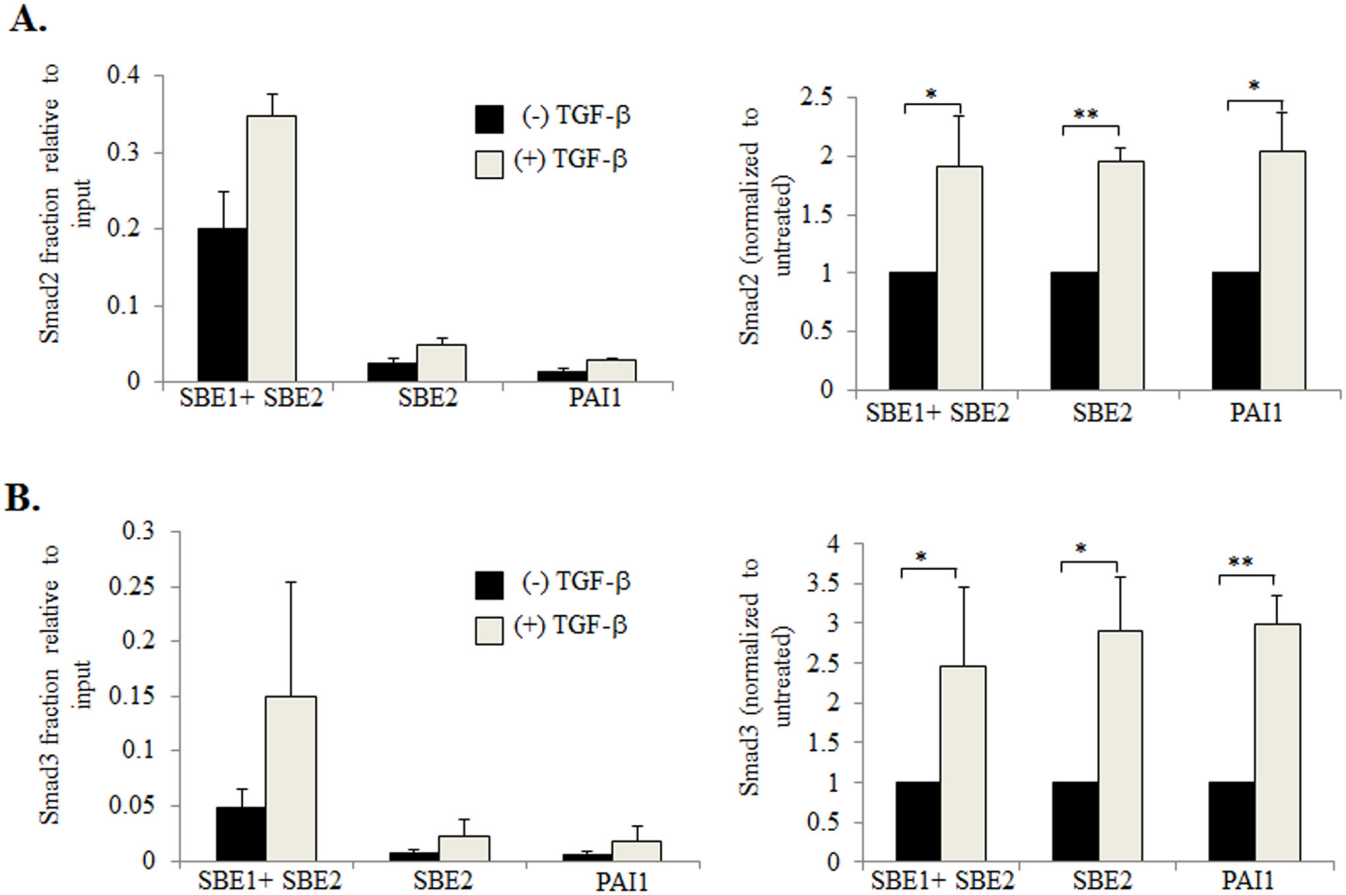
TGF-β enhances binding of Smad2 and Smad3 to the CXCL1 promoter. Genomic DNA was isolated from 41CAFs treated with 5 ng/ml of TGF-β for 6 hours, and immunoprecipated with: control IgG, or antibodies to Smad2 or Smad3. Samples were analyzed by real-time PCR analysis for (A) Smad2 or (B) Smad3 binding to SBE1 and SBE2 or SBE2 alone on the CXCL1 promoter. Smad2/3 binding to the PAI1 promoter was evaluated as a positive control for TGF-β responsiveness. Background from IgG control was subtracted from samples. Left panels show fraction of Smad2 or 3 binding to DNA relative to input control. Right panel shows Smad2 or Smad3 binding normalized to (-) TGF-β group for each promoter region. Statistical analysis was performed using Two Tailed T-test. Statistical significance was determined by p<0.05; *p<0.05, **p<0.01. Values are expressed as Mean ± SEM.

Chromatin Immunoprecipitation assays (ChIP) were performed to determine whether Smad2/3 proteins bound to these putative SBEs in the CXCL1 promoter. One set of primers were designed to amplify a region containing both SBEs (SBE1+SBE2). A second set of primers were designed to amplify a region containing only SBE2, enabling us to determine the relative importance of both SBEs to Smad2/3 binding. To perform the ChIP assays, genomic DNA samples were isolated from 41CAFs cultured in the presence or absence of TGF-β. DNA samples were bound to antibodies to Smad2 or Smad3 or IgG control, and subject to PCR amplification using: SBE1+SBE2 or SBE2 primers. The PAI1 promoter was subject to PCR amplication, as a positive control for TGF-β responsiveness. In the absence of TGF-β treatment, samples bound to Smad2 antibodies showed detectable amplicon levels, when subject to PCR with primers amplifying SBE1+SBE2, or only SBE2. Expression levels were amplified 2 fold with TGF-β treatment (Fig 2A). We also observed a significant increase in amplicon levels with TGF-β treatment in samples bound to Smad3 antibodies (Fig 2B). Samples amplifying SBE2 alone showed similar expression levels to the positive control. Expression levels were noticeably higher in samples amplifying SBE1+SBE2, compared to samples with SBE2 alone. Taken together, these results indicate that in the absence of TGF-β, Smad2/3 proteins bind to both SBEs identified in the CXCL1 promoter, and that TGF-β treatment promotes additional Smad2/3 binding to these elements.

**Fig 3:**
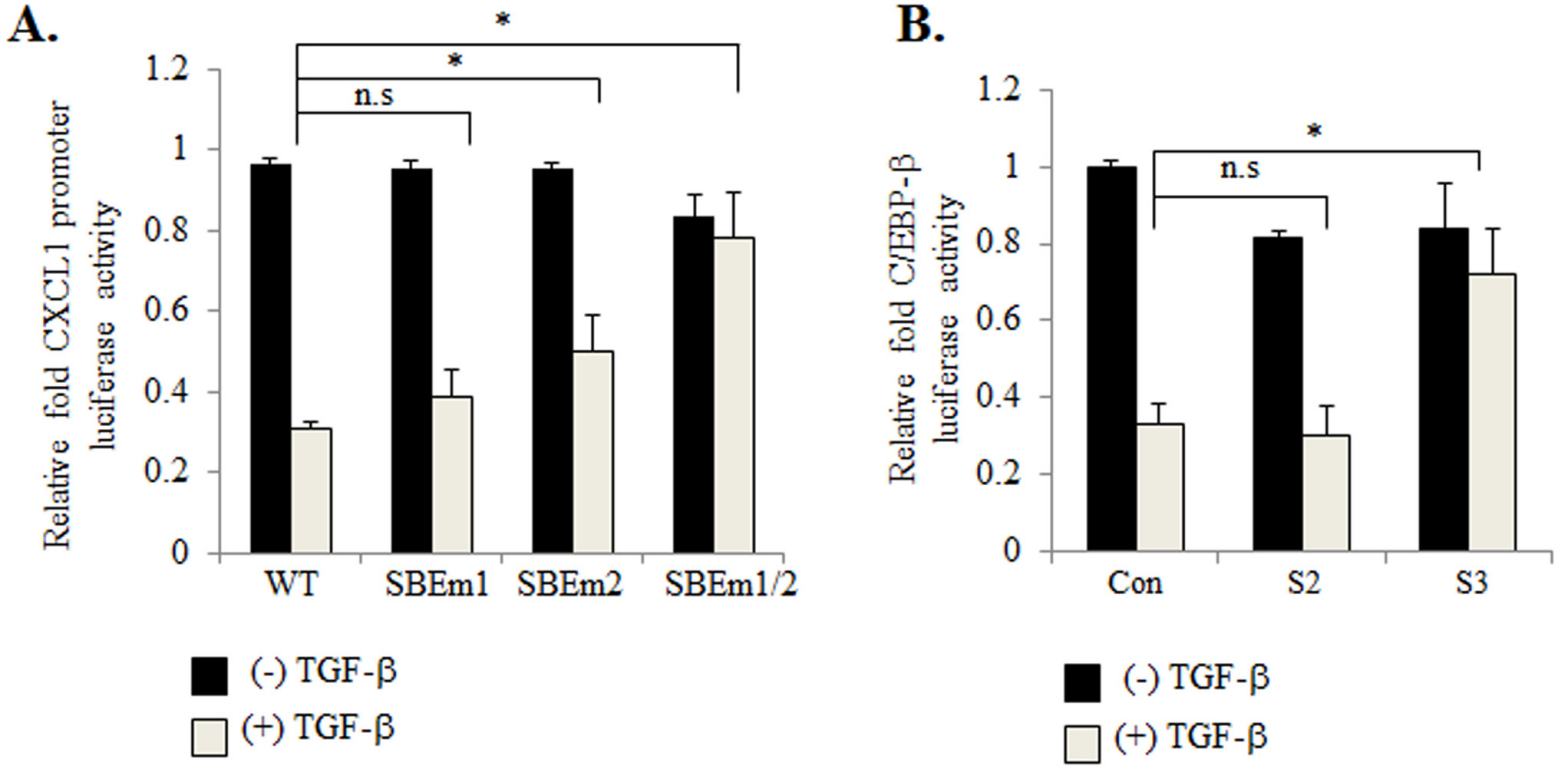
Smad2 and Smad3 modulate CXCL1 promoter activity through C/EBP-β dependent and independent mechanisms. (A) 41CAFs were co-transfected with Renilla luciferase and firefly luciferase reporters containing: the wildtype CXCL1 promoter (PGL3.luc.CXCL1), or mutations in SBE1 (SBEm1), SBE2 (SBEm2), or both (SBEm1/2). Cells were treated with 5 ng/ml of TGF-β for 6 hours, and analyzed for CXCL1 promoter activity by luciferase assay. Fold change was calculated relative to (-) TGF-β group. (B) 41CAFs co-expressing C/EBP-β.luc firefly and Renilla luciferase reporters were transfected with: control siRNA (Cont) or siRNAs to Smad2 (S2), Smad3 (S3). Cells were treated with 5 ng/ml of TGF-β for 24 hours and C/EBP-β activity by luciferase assay. Fold change was calculated relative to control siRNA/(-) TGF-β group. Firefly luciferase values were normalized to Renilla luciferase. Statistical analysis was performed using One Way ANOVA followed by Bonferonni post-hoc comparisons. Statistical significance was determined by p<0.05; *p<0.05, n.s; not significant. Values are expressed as Mean ± SEM.

To determine whether these SBEs were important for CXCL1 expression, inactivating point mutations were introduced into each of the SBEs in the CXCL1 promoter driving firefly luciferase expression. 41CAFs were co-transfected with Renilla luciferase reporter, and a firefly luciferase reporter containing the CXCL1 promoter with wildtype (WT) SBE, mutant SBE1 or mutant SBE2. Cells were treated with TGF-β, and then analyzed for CXCL1 promoter activity by luciferase assay. TGF-β treated cells that were transfected with mutant SBE1, showed a modest increase in luciferase activity, compared to cells with WT SBE. TGF-β treated cells that were transfected with mutant SBE2, showed a larger increase in luciferase activity compared to cells with WT SBE. TGF-β treated cells with mutations in both SBEs showed the largest increase in luciferase activity (Fig 3A). These data indicate that both SBEs are important for TGF-β repression of CXCL1 promoter activity.

### Smad3 Represses C/EBP-β Transactivation

As Smad2/3 proteins bound to the CXCL1 promoter without a TIE sequence, we hypothesized that Smad2/3 proteins would inhibit CXCL1 gene transcription by blocking activity of adjacent co-factors. By candidate screening, we identified C/EBP-β as a possible co-factor positively regulating CXCL1 transcription in mammary CAFs. Smad3 proteins have been shown to complex with and repress C/EBP-β transcriptional activity in astrocytes, 3T3 fibroblasts and adipocytes. Studies have further shown that Smad3 can interact with, and interfere with the ability of C/EBP-β to bind to the promoter [40–42]. In addition, a C/EBP-β binding motif (5’-TGGAGCAAG-3’) was identified at position -128 to -120 in the mouse CXCL1 promoter, proximal to the SBEs. To determine whether Smad2 or Smad3 were important for regulating C/EBP-β activity in CAFs, 41CAFs co-expressing Renilla luciferase and C/EBP-β firefly luciferase reporter plasmids, were transfected with control siRNAs, or siRNAs to Smad2, Smad3 or both. The cells were treated with TGF-β, and then analyzed for changes in C/EBP-β promoter activity by luciferase assay. 41CAFs expressing control siRNAs showed detectable C/EBP-β promoter activity, which was inhibited by TGF-β treatment (Fig 3B). Knockdown of Smad3, but not Smad2 increased C/EBP-β promoter activity in cells treated with TGF-β, compared to control siRNA expressing cells (Fig 3B). Taken together, these data indicate that Smad3 is important for C/EBP-β activity in 41CAFs.

### HGF Antagonizes TGF-β Signaling in CAFs to Positively Regulate CXCL1 Expression

**Fig 4:**
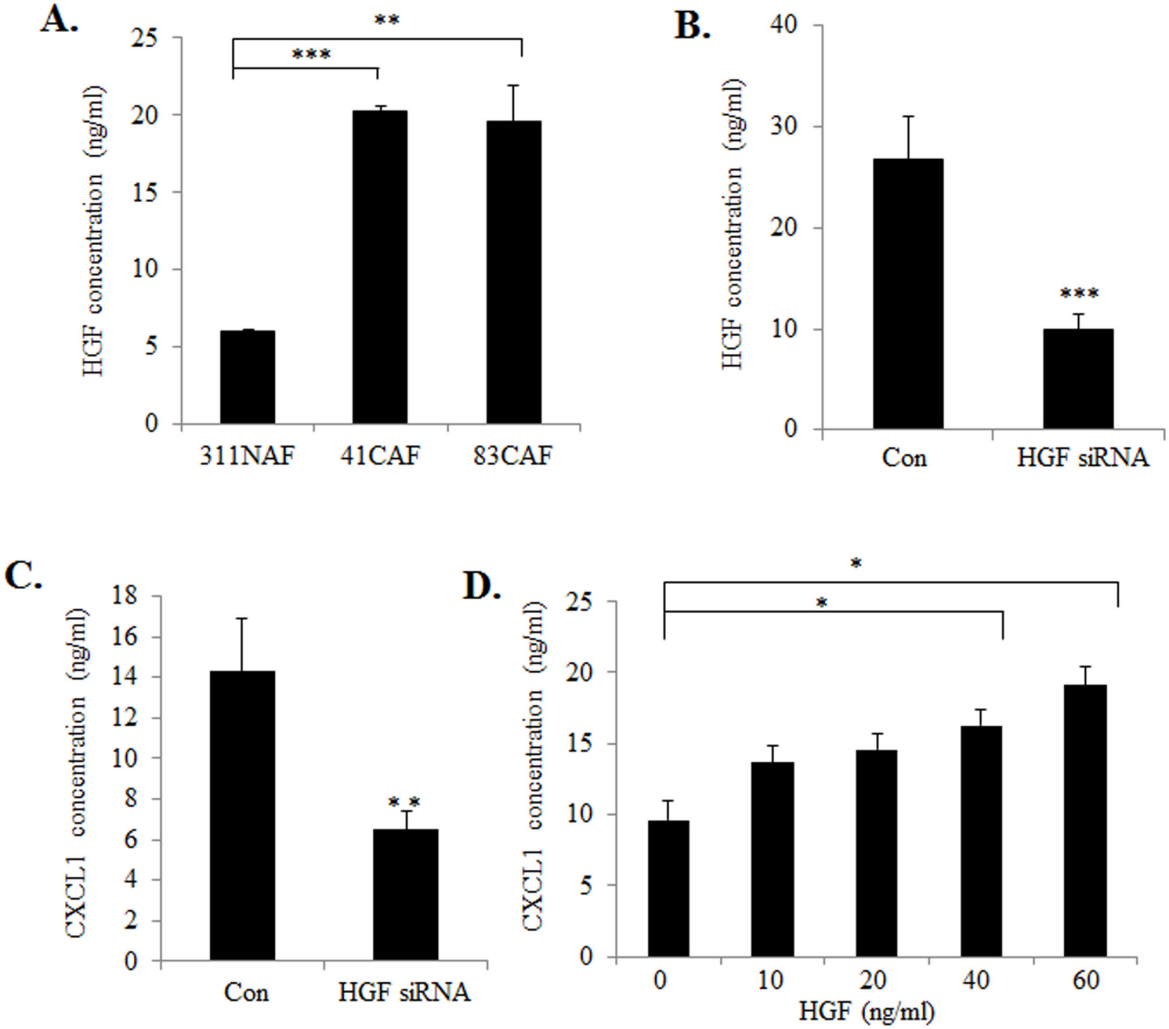
HGF positively regulates CXCL1 expression in mammary fibroblasts. (A) The indicated cell lines were analyzed for HGF expression in conditioned media by ELISA. (B-C) 41CAFs expressing control siRNA (Con) or siRNAs to HGF were analyzed for expression of HGF (B) or CXCL1 (C) by ELISA. (D) 41CAFs were treated with increasing concentrations of HGF for 48 hours and analyzed for CXCL1 expression by ELISA. Statistical analysis was performed using Two Tailed T-Test (B,C), or One Way ANOVA followed by Bonferonni post-hoc comparisons (A,D). Statistical significance was determined by p<0.05; *p<0.05, **p<0.01, ***p<0.001. Values are expressed as Mean ± SEM.

In addition to decreased TGF-β expression, breast cancer stroma also show increased expression of HGF, a growth factor that acts on breast cancer cells to promote tumor growth and invasion [6, 43]. Previous studies demonstrated that TGF-β negatively regulates expression of HGF in fibroblasts [44]. We therefore hypothesized that TGF-β suppressed CXCL1 expression through a secondary mechanism related to HGF signaling. We first determined whether HGF was important for regulating CXCL1 expression in CAFs. Consistent with previous studies of human CAFs, HGF expression was elevated in mouse CAF lines compared to NAFs, corresponding to increased CXCL1 expression (Fig 4A). siRNA knockdown of HGF in 41CAFs resulted in a partial, but statistically significant reduction of CXCL1 protein expression, corresponding to decreased HGF expression levels (Fig 4B and 4C). Furthermore, HGF treatment of 41CAFs enhanced CXCL1 expression in a dose dependent manner (Fig 4D). These data indicate that HGF positively regulates CXCL1 expression in CAFs.

**Fig 5:**
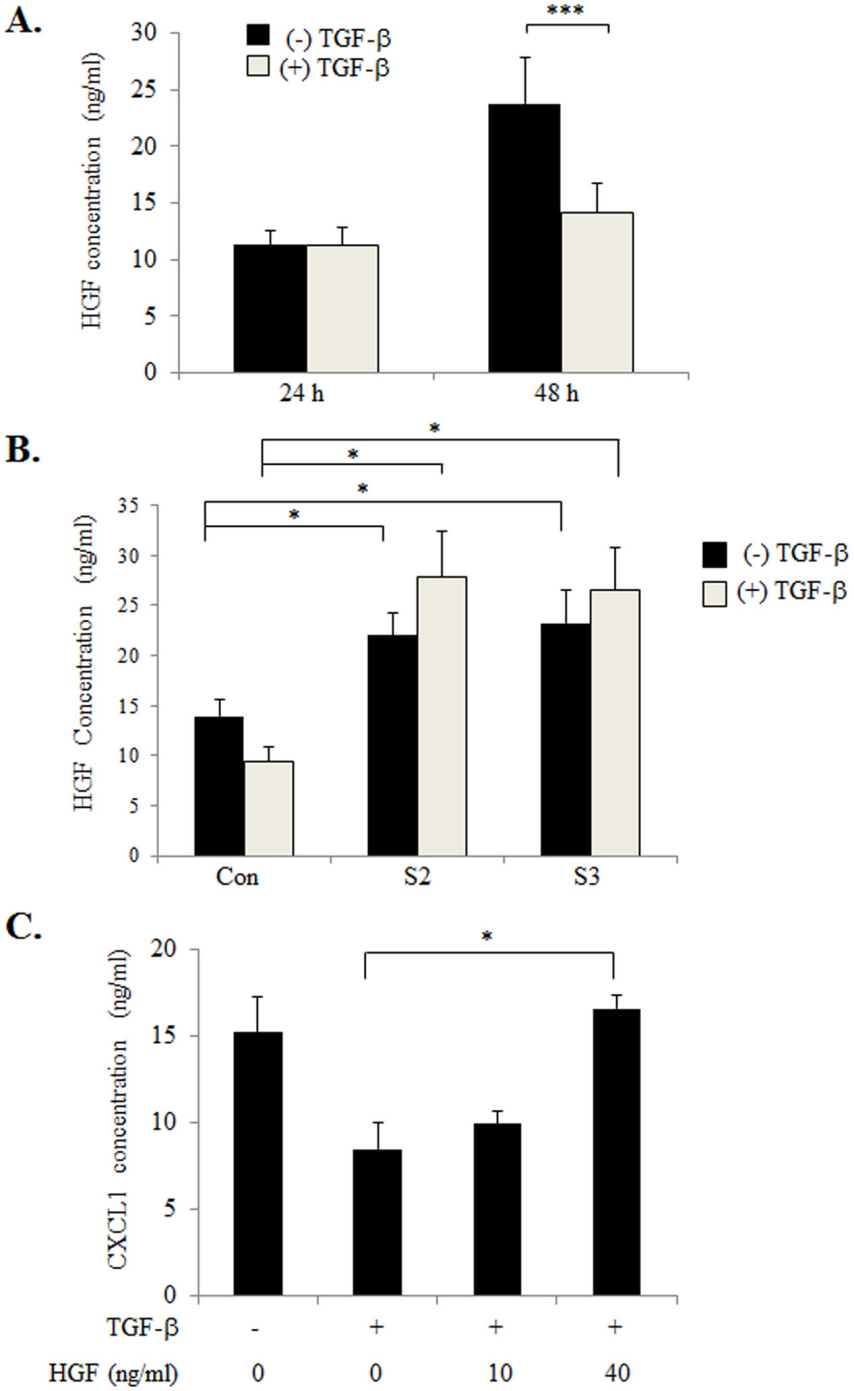
TGF-β inhibits HGF expression through Smad2/3 dependent mechanisms. (A) 41CAFs were treated with 5ng/ml TGF-β for 24 or 48 hours, and analyzed for HGF expression in conditioned media by ELISA. (B) 41CAFs were transfected with control siRNA (Con), or siRNAs to Smad2 (S2) or Smad3 (S3), treated with 5 ng/ml TGF-β for 48 hours, and analyzed for HGF expression in by ELISA. (C) 41CAFs were treated with 5ng/ml TGF-β in the presence or absence of increasing concentrations of HGF for 48 hours, and analyzed for CXCL1 expression by ELISA. Statistical analysis was performed using Two Tailed T-Test (A), or One Way ANOVA followed by Bonferonni post-hoc comparisons (B, C). Statistical significance was determined by p<0.05; *p<0.05, ***p<0.001. Values are expressed as Mean ± SEM.

We next determined whether TGF-β expression modulated HGF expression in 41CAFs. TGF-β treatment inhibited HGF expression as determined by ELISA. siRNA knockdown of Smad2 or Smad3 in 41CAFs alone resulted in increased HGF expression. Knockdown of Smad2 or Smad3 also inhibited TGF-β responsiveness, sustaining levels of HGF expression (Fig 5A and 5B). These data indicated that TGF-β negatively regulated HGF expression through Smad2/3 dependent mechanisms. While TGF-β inhibited CXCL1 expression in 41CAFs, the addition of HGF to TGF-β treated 41CAFs, increased CXCL1 expression (Fig 5C), indicating that HGF prevents TGF-β from suppressing CXCL1 expression in fibroblasts. Taken together, these studies demonstrate TGF-β and HGF negatively interact with other to modulate CXCL1 expression in 41CAFs.

### c-Met Receptor Tyrosine Kinases Modulate CXCL1 Expression in CAFs

**Fig 6:**
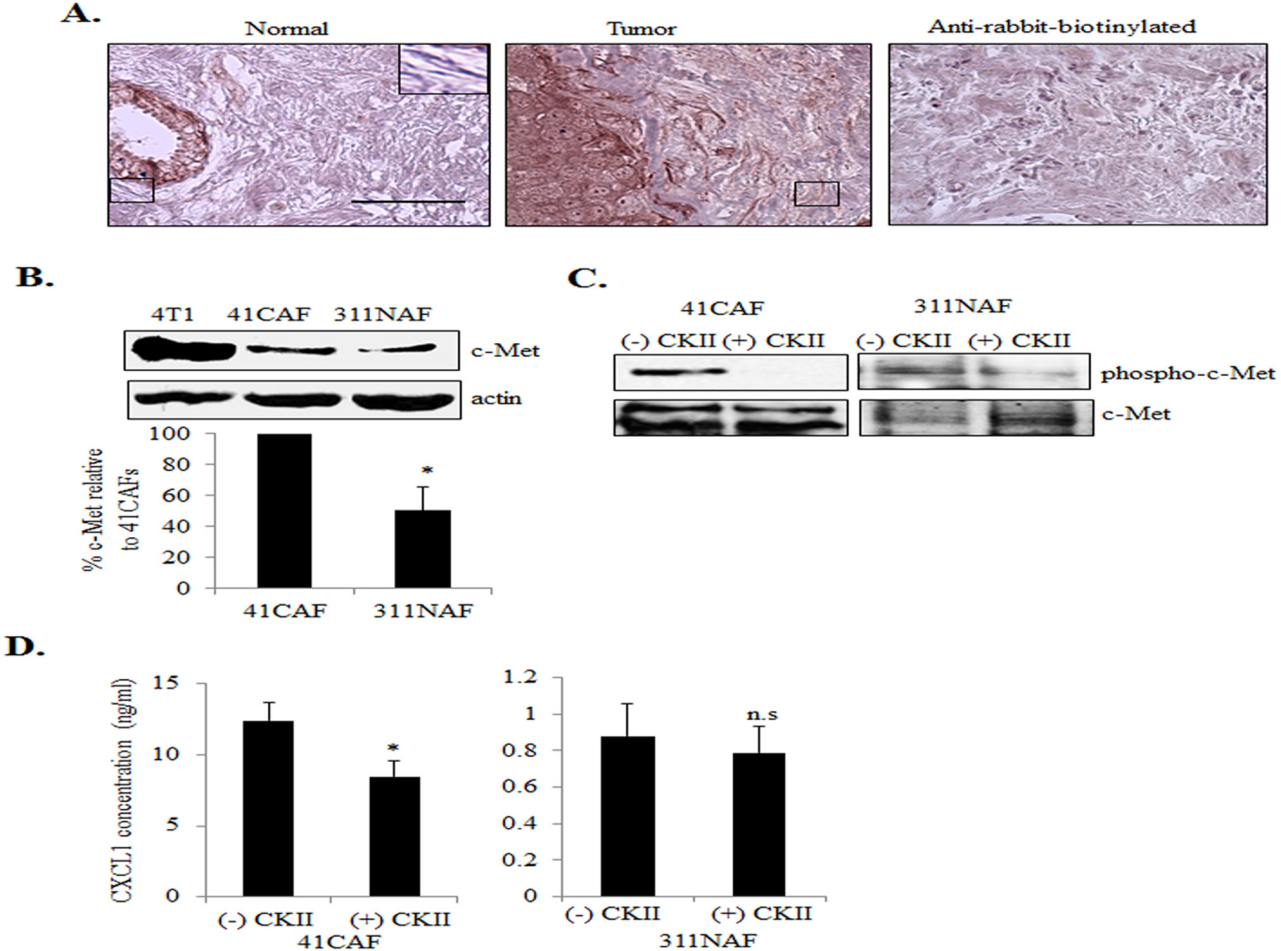
c-Met receptor tyrosine kinases positively regulate CXCL1 expression in mammary CAFs. (A) Normal breast (n = 3) or breast tumor tissues (n = 4) were immunostained for c-Met expression. Magnified inset shows representative staining in fibroblasts. E = epithelium, BV = blood vessel. Scale bar = 50 microns. Secondary antibody control = anti-rabbit-biotinylated. (B) 311NAFs and 41CAFs were analyzed for c-Met expression by immunoblot analysis. c-Met expression was normalized to actin by densitometry analysis. 4T1 mammary carcinoma cells are shown as a positive control. (C) 41CAFs and 311 NAFs were treated with 200 nM c-Met kinase inhibitor type II (CKII) for 1 hour, and analyzed for expression of phospho-c-Met (Tyr-1234/1235) by immunoblot. (D) 41CAFs or 311NAFs treated with CKII for 48 hours were analyzed for CXCL1 expression by ELISA. Statistical analysis was performed using Two Tailed T-Test. Statistical significance was determined by p<0.05; *p<0.05, n.s; not significant. Values are expressed as Mean ± SEM.

HGF primarily binds to c-Met receptor tyrosine kinases, which are thought to be primarily expressed in epithelial cells [45]. However, c-Met has been detected in sclerotic and osteoarthritis synovial fibroblasts, indicating that c-Met may be expressed in mesenchymal cells under pathological conditions [46, 47]. We first analyzed for receptor expression in normal and breast ductal carcinomas by immunohistochemistry. c-Met expression was detected in fibroblastic cells of breast tumor tissues. Fibroblasts in normal breast tissues showed low to undetectable levels of c-Met expression (Fig 6A). Expression of c-Met in fibroblasts was verified by co-immunofluoresence staining for c-Met with α-smooth muscle actin (α-sma) (S2 Fig), a marker for activated fibroblasts [48, 49]. We then analyzed for c-Met expression in mammary fibroblast lines by immunoblot, using 4T1 mammary carcinoma cells as a positive control. By densitometry analysis, 41CAFs showed significantly higher expression of c-Met compared to 311NAFs (Fig 6B). 41CAFs showed detectable levels of c-Met phosphorylation at Tyr1234/1235 (Fig 6C), sites critical for receptor activation [50, 51]. Treatment with the c-Met pharmacologic inhibitor CKII [IC50 = 200 nM [52, 53]] reduced phosphorylation of c-Met, and decreased CXCL1 expression in 41CAFs. Interestingly, CKII treatment also reduced expression of phosphorylated c-Met, but did not significantly affect CXCL1 expression in 311NAFs (Fig 6C and 6D). To determine whether c-Met activity was restricted to one cell line, we evaluated other fibroblast lines. 83CAFs also showed increased c-Met expression compared to 311NAFs, and showed a reduction in CXCL1 expression with CKII treatment (S3 Fig). CKII treatment inhibited CXCL1 expression in cultured human CAFs (S4 Fig). These studies indicate that regulation of CXCL1 expression through c-Met is a mechanism present in multiple CAF lines.

### HGF/c-Met Signaling Regulates CXCL1 Expression through NF-κB Dependent Mechanisms

**Fig 7:**
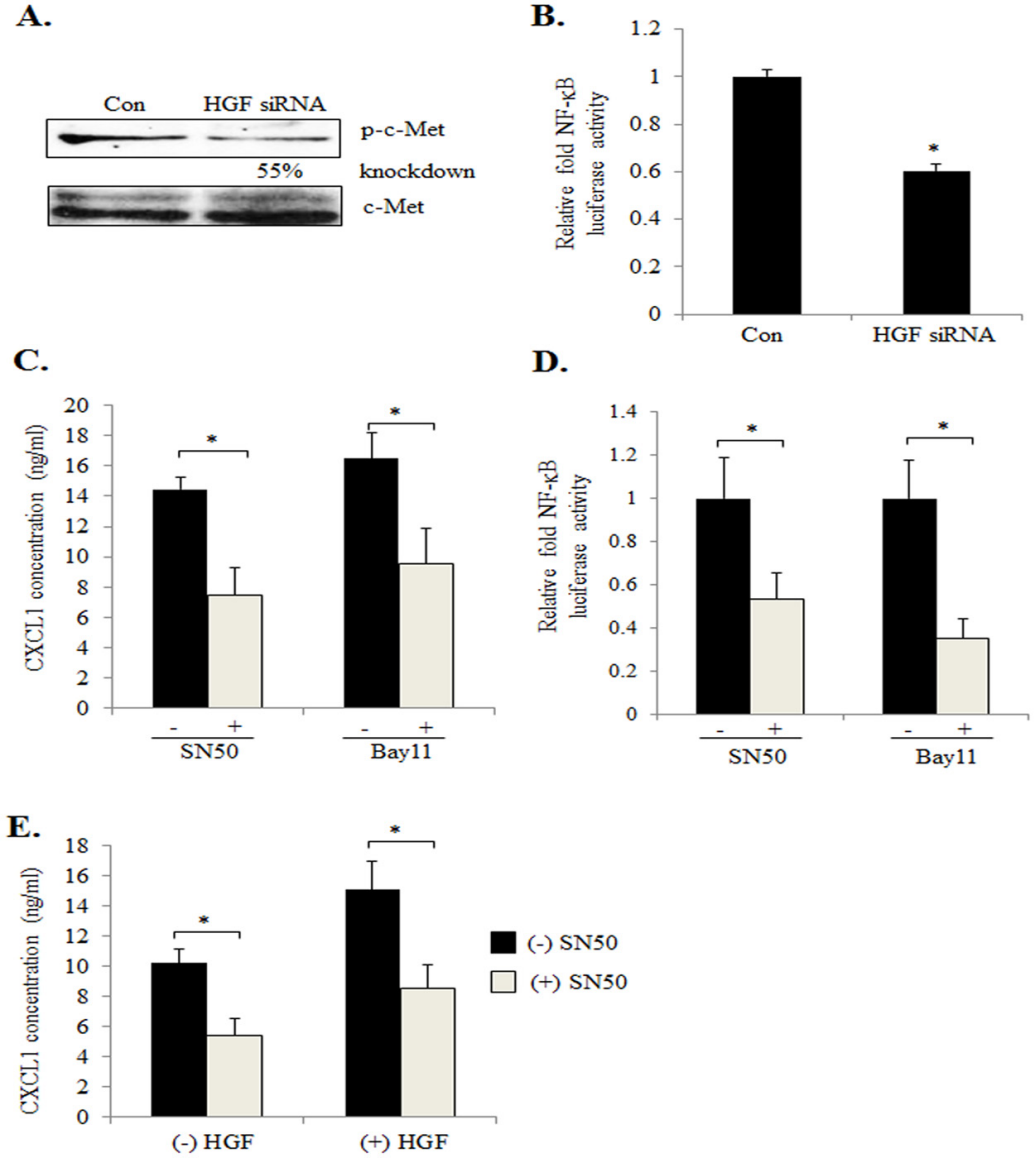
HGF/c-Met signaling regulates CXCL1 expression in mammary CAFs through NF-κB dependent mechanisms. (A) 41CAFs were transfected with control or HGF siRNAs, and analyzed for expression of phospho-c-Met (Tyr-1234/1235) by immunoblot. Expression of phospho-c-Met was normalized to total c-Met by densitometry analysis. (B) 41CAFs co-expressing pNF-κB.luc and Renilla luciferase reporters were transfected with control (Con) or HGF siRNAs, and analyzed for NF-κB activity by luciferase assay. Fold change was calculated relative to control siRNA group. (C) 41CAFs were treated with 36 μM SN50 or 5 μM Bay11-7085 for 24 hours, and analyzed for CXCL1 expression by ELISA. (D) 41CAFs co-expressing pNF-κB.luc and Renilla luciferase reporters were treated with 36 μM SN50 or 5 μM Bay 11–7085, and analyzed for NF-κB activity by luciferase assay. Firefly luciferase values were normalized to Renilla luciferase. Fold change was calculated relative to (-) SN50 or (-) Bay 11–7085 group. (E) 41CAFs were treated with 40 ng/ml HGF in the presence or absence of 36 μM SN50 for 24 hours, and then analyzed for CXCL1 expression by ELISA. Statistical analysis was performed using Two Tailed T-Test. Statistical significance was determined by p<0.05; *p<0.05. Values are expressed as Mean ± SEM.

We next determined which pathways would be important for HGF positive regulation of CXCL1 expression. HGF has been shown to regulate activity of NF-κB in epithelial cells [54–56], and two NF-κB binding sites have been identified in the CXCL1 promoter [57]. To determine the importance of HGF/c-Met signaling in regulating NF-κB activity in mammary CAFs, 41CAFs were transfected with control or HGF siRNAs. HGF knockdown decreased phospho-c-Met expression, relative to control cells (Fig 7A), and significantly reduced NF-κB activity, as determined by luciferase assay (Fig 7B). To determine the significance of NF-κB to CXCL1 expression, 41CAFs were treated with SN50, an peptide inhibitor that blocks NF-κB translocation to the nucleus [IC50 = 20 μ**M**, [58]], and Bay 11–7085, a pharmacologic inhibitor that blocks IκB phosphorylation [IC50 = 10 μM, [59]]. We observed significantly reduced CXCL1 protein expression in 41CAFs, corresponding to reduced NF-κB activity (Fig 7C and 7D). Treatment of 83CAFs with SN50 and Bay 11–7085 also decreased CXCL1 expression (S5 Fig). To determine whether NF-κB was important in HGF induction of CXCL1 expression, 41CAFs were treated with HGF in the presence or absence of SN50. By ELISA, SN50 treatment significantly inhibited the levels of CXCL1 expression in HGF treated cells (Fig 7E). These data indicate that HGF/c-Met signaling positively regulates CXCL1 expression in mammary CAFs through an NF-κB dependent manner.

## Discussion

Overexpression of CXCL1 in the breast cancer stroma is associated with poor patient prognosis [20]. Here, we used a combination of siRNA and pharmacologic approaches to demonstrate that key factors expressed in breast cancer, TGF-β and HGF, modulate CXCL1 expression in CAFs. TGF-β signaling activates Smad2/3 proteins, which bind to elements on the CXCL1 promoter, and inhibit gene expression. In a secondary mechanism, TGF-β inhibits CXCL1 expression by down-regulating expression of HGF, which signals to c-Met to positively regulate CXCL1 expression through NF-κB dependent mechanisms.

While NAFs and CAFs exhibit similar cell morphologies, co-transplantation studies have demonstrated that CAFs enhance tumor growth, survival and invasiveness, corresponding to increased expression of growth factors, angiogenic factors and cytokines [3]. However, the mechanisms that regulate tumor promoting factors in CAFs remain poorly understood. Here, we observed some similarities and differences in the way that CXCL1 expression is regulated in CAFs and NAFs. Exogenous TGF-β inhibited CXCL1 expression in both NAFs and CAFs. However, CAFs showed decreased endogenous TGF-β compared to NAFs [20]. HGF and c-Met were also increased in CAFs compared to NAFs, and CKII treatment reduced CXCL1 expression in CAFs, but not NAFs, indicating increased HGF/c-Met autocrine signaling in CAFs. We also observed that HGF treatment of CAFs prevented TGF-β from suppressing CXCL1 expression. While TGF-β and HGF are both co-expressed in the tumor [22, 60], our studies indicate that low TGF-β concentrations and high HGF concentrations in the local breast cancer stroma may result in increased CXCL1 expression.

Here, we show that TGF-β suppresses CXCL1 expression through Smad2/3 dependent mechanisms in mammary CAFs. Knockdown of both Smad2 and3 significantly inhibited TGF-β responsiveness and significantly enhanced CXCL1 promoter activity and protein expression, compared to knockdown of Smad2 or Smad3 alone. While Smad2 and Smad3 may function independently to modulate gene expression and TGF-β responsiveness [61, 62] our data suggest that cooperation of Smad2 and Smad3 are important in TGF-β mediated repression of CXCL1 expression. Interestingly, we noted that Smad3, but not Smad2 was required for repression of C/EBP-β, a transactivator of CXCL1 expression. These data indicate that Smad2 inhibits CXCL1 promoter activity through an additional mechanism, possibly inhibiting CXCL1 promoter activity through other cooperating factors, independent of Smad3. Interestingly, Smad2 may be expressed as two different splice variants. One splice variant of Smad2 is more prevalent in mammalian cells [63], and contains an insert in the MH1 domain, which is encoded by exon 3 and prevents DNA binding [38, 64]. The other variant, commonly referred to as Smad2Δexon3, lacks the insert, and is able to bind DNA to activate gene transcription [64]. Both variants are capable of forming complexes with Smad3 and Smad4 [64]. It is possible that both Smad2 variants are involved in regulating CXCL1 expression, as the siRNA targeting sequences were not specific to one variant. Smad2/4 complexes interact with a variety of transcription factors, including Sp-1, Fast-1, Mixer and Milk [65, 66]. Investigation of this mechanism would involve characterization of the Smad2 gene variant in mammary CAFs, and pull-down experiments to identify interacting transcription factors, experiments beyond the scope of this report.

In our studies, we identified two new SBE sites in the CXCL1 promoter. Our ChIP studies indicated that more Smad2/3 proteins bound to the CXCL1 promoter region encompassing both SBEs, compared to the region encompassing SBE2 alone. Interestingly, mutational analysis of the SBEs showed that TGF-β responsiveness is not affected by mutation of SBE1 alone. However, there is a strong possibility that that SBE1 is a functioning Smad binding element. SBE1 and SBE2 have identical sequences for Smad2 and Smad3 binding, as identified from previous studies [38, 39]. Furthermore, mutation of both SBE1 and SBE2 abrogated TGF-β suppression of CXCL1 promoter activity. On-going studies in our lab indicate that CCL2 chemokine signaling inhibits CXCL1 promoter activity, and that mutation of SBE1 inhibits CCL2 mediated suppression of CXCL1 promoter activity (unpublished data). These data are consistent with our previous studies showing that CCL2 is capable of activating Smad3, independent of TGF-β [67]. Taken together, these data suggest that SBE1 is a functioning Smad binding element. In the context of TGF-β signaling, SBE1 alone may not be sufficient to modulate TGF-β suppression of CXCL1 promoter activity. Both SBEs may be important in recruiting sufficient levels of Smad2 and Smad3 proteins to suppress CXCL1 gene expression.

At this time, it is unclear how Smad binding to SBE1 and SBE2 functions to modulate CXCL1 promoter activity. We examined the possibility that Smad2 and Smad3 might recruit other transcription factors to bind to the CXCL1 promoter region between -271 to -123 that encompassed both SBE1 and SBE2 in our ChIP studies. NF-κB, Stat1, poly(ADP-ribose) polymerase-1, High Mobility Group proteins, Sp1 and mutant p53 bind to the CXCL1 promoter. However, these transcription factors positively regulate expression and bind outside the region -271 to -123 [68–71]. We do not exclude the possibility that Smad2 or Smad3 may recruit other transcriptional repressors to the CXCL1 promoter. Such studies would involve transcription factor profiling and biochemical studies, which are of interest for the future.

Through a secondary mechanism, we show that TGF-β negatively regulates CXCL1 expression by down-regulating HGF expression. Multiple studies have demonstrated that HGF is overexpressed in breast cancer stroma, and binds to c-Met receptors expressed on epithelial cells to regulate cancer progression [43, 72–74]. Our studies are the first to report an important role for HGF/c-Met autocrine signaling in mammary CAFs in regulating gene expression. It is possible that c-Met receptor expression in the stroma may have been overlooked in previously published studies, as c-Met expression is lower in fibroblasts compared to mammary carcinoma cells. Here, we show that expression of c-Met is increased in CAFs compared to NAFs, consistent with previous studies showing positive c-Met expression in fibroblasts under pathological conditions, such as systemic sclerosis and osteoarthritis [46, 47]. CKII inhibits CXCL1 expression in CAFs, corresponding to decreased expression of phosphorylated c-Met protein. Interestingly, CKII treatment reduced expression of phosphorylated c-Met in NAFs, but did not significantly affect CXCL1 expression. As HGF expression is lower in NAFs compared to CAFs, the levels of c-Met receptor activity in NAFs may not be important in modulating CXCL1 expression. These data suggest that levels of the c-Met receptor and HGF ligand are important for positive regulation of CXCL1 expression in mammary fibroblasts.

## Conclusions

In summary, CAFs overexpress CXCL1, a chemokine that promotes the progression and drug resistance of breast tumors. We illustrate important molecular mechanisms modulating CXCL1 expression in mammary CAFs. By understanding the molecular mechanisms of oncogene expression in CAFs, we may better define the role of fibroblasts in tumor progression.

## Supporting Information

S1 Fig Smad2 and Smad3 knockdown in 83CAFs inhibit TGF-β responsiveness and elevate CXCL1 expression. 83CAFs were transfected with control, Smad2 or Smad3 siRNAs, or both, and treated with TGF-β for 24 hours. CXCL1 expression was determined by ELISA. Statistical analysis was performed using One Way ANOVA followed by Bonferonni post-hoc comparisons. Statistical significance was determined by p<0.05; *p<0.05, n.s; not significant. Values are expressed as Mean ± SEM.
(TIF)

S2 Fig c-Met receptors co-localize with α-sma in breast tumor tissues. Normal breast tissues or breast carcinoma tissues from patient samples were immunofluorescence stained with antibodies to c-Met (red) and α-sma) (green). PyVmT mammary carcinoma tissues were used as a positive control. Arrows point to c-Met co-localization with α-sma. Sections were counterstained with DAPI. Secondary antibody controls are shown: anti-rabbit-Alexa-568 for c-Met, and anti-mouse-biotinylated conjugated to streptavidin-Alexa-488 for α-sma. Scale bar = 100 microns.
(TIF)

S3 Fig c-Met inhibitors reduce CXCL1 expression in 83CAFs. (A) 83CAFs and 311NAFs were analyzed for c-Met expression by immunoblot analysis. c-Met expression was normalized to actin by densitometry analysis. (B) 83CAFs were treated with 200 nM CKII for 48 hours, and analyzed for CXCL1 expression by ELISA. Statistical analysis was performed using Two Tailed T-Test. Statistical significance was determined by p<0.05; *p<0.05. Values are expressed as Mean ± SEM.
(TIF)

S4 Fig c-Met inhibitors reduce CXCL1 expression in human CAFs. Human CAFs were treated with 200 nM CKII for 48 hours and analyzed for CXCL1 expression by ELISA. Statistical analysis was performed using Two Tailed T-Test. Statistical significance was determined by p<0.05; *p<0.05. Values are expressed as Mean ± SEM.
(TIF)

S5 Fig NF-κB inhibitors reduce CXCL1 expression in 83CAFs. 83CAFs were treated with 36 μM SN50 and 5 μM Bay11-7085 for 24 hours, and analyzed for CXCL1 expression by ELISA. Statistical analysis was performed using Two Tailed T-Test. Statistical significance was determined by p<0.05; *p<0.05. Values are expressed as Mean ± SEM.
(TIF)
