# Supplementary figures and images for: TGF-β Negatively Regulates CXCL1 Chemokine Expression in Mammary Fibroblasts through Enhancement of Smad2/3 and Suppression of HGF/c-Met Signaling Mechanisms
Source: PLoS One. 2015 Aug 7;10(8):e0135063. doi: 10.1371/journal.pone.0135063 (PMC4529193; doi:10.1371/journal.pone.0135063)

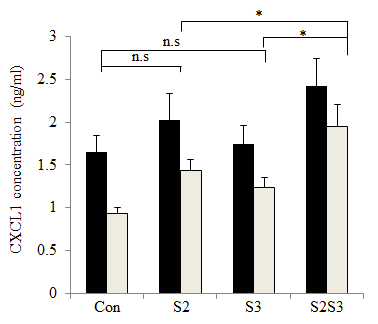

Supplement: S1 Fig — 83CAFs were transfected with control, Smad2 or Smad3 siRNAs, or both, and treated with TGF-β for 24 hours. CXCL1 expression was determined by ELISA. Statistical analysis was performed using One Way ANOVA followed by Bonferonni post-hoc comparisons. Statistical significance was determined by p<0.05; *p<0.05, n.s; not significant. Values are expressed as Mean ± SEM. (TIF) [file pone.0135063.s001.tif]

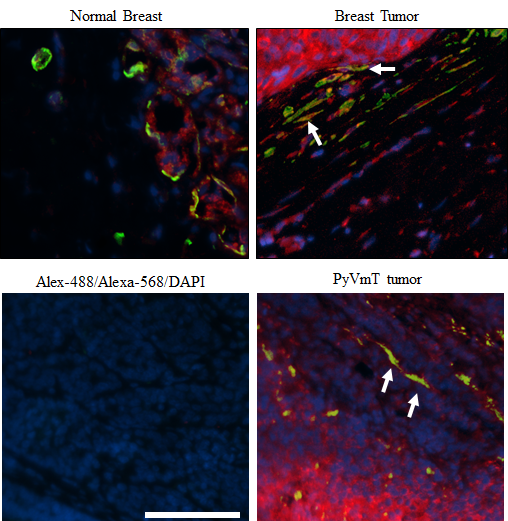

Supplement: S2 Fig — Normal breast tissues or breast carcinoma tissues from patient samples were immunofluorescence stained with antibodies to c-Met (red) and α-sma) (green). PyVmT mammary carcinoma tissues were used as a positive control. Arrows point to c-Met co-localization with α-sma. Sections were counterstained with DAPI. Secondary antibody controls are shown: anti-rabbit-Alexa-568 for c-Met, and anti-mouse-biotinylated conjugated to streptavidin-Alexa-488 for α-sma. Scale bar = 100 microns. (TIF) [file pone.0135063.s002.tif]

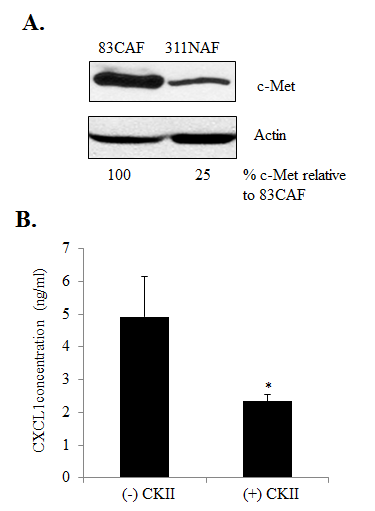

Supplement: S3 Fig — (A) 83CAFs and 311NAFs were analyzed for c-Met expression by immunoblot analysis. c-Met expression was normalized to actin by densitometry analysis. (B) 83CAFs were treated with 200 nM CKII for 48 hours, and analyzed for CXCL1 expression by ELISA. Statistical analysis was performed using Two Tailed T-Test. Statistical significance was determined by p<0.05; *p<0.05. Values are expressed as Mean ± SEM. (TIF) [file pone.0135063.s003.tif]

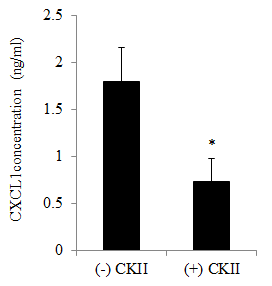

Supplement: S4 Fig — Human CAFs were treated with 200 nM CKII for 48 hours and analyzed for CXCL1 expression by ELISA. Statistical analysis was performed using Two Tailed T-Test. Statistical significance was determined by p<0.05; *p<0.05. Values are expressed as Mean ± SEM. (TIF) [file pone.0135063.s004.tif]

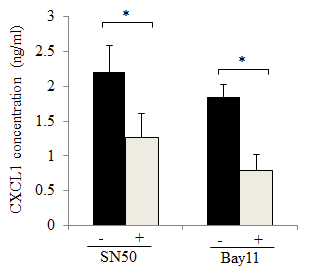

Supplement: S5 Fig — 83CAFs were treated with 36 μM SN50 and 5 μM Bay11-7085 for 24 hours, and analyzed for CXCL1 expression by ELISA. Statistical analysis was performed using Two Tailed T-Test. Statistical significance was determined by p<0.05; *p<0.05. Values are expressed as Mean ± SEM. (TIF) [file pone.0135063.s005.tif]
